# The seen and be heard study: A national mixed methods study to identify the barriers and facilitators to ensuring equitable cancer care for children with and without learning disabilities and/or who are autistic – Protocol Paper

**DOI:** 10.1371/journal.pone.0333020

**Published:** 2025-11-19

**Authors:** Kate Oulton, Jo Wray, Mary Foo-Caballero, Samantha Flynn, Phillip Harniess, Anupama Rao, Faith Gibson

**Affiliations:** 1 Centre for Outcomes and Experience Research in Children’s Health, Illness and Disability (ORCHID), Zayed Centre for Research, Great Ormond Street Hospital for Children NHS Foundation Trust, London, England; 2 Great Ormond Street Hospital for Children NHS Foundation Trust, London, England; 3 Centre for Educational Development, Appraisal and Research, The University of Warwick, Warwick, England; 4 Peninsula Childhood Disability Research Unit (PenCRU) and NIHR Applied Research Collaboration South-West Peninsula (PenARC), University of Exeter Medical School, University of Exeter, Exeter, England; 5 School of Health Sciences, University of Surrey, Guildford, England; Hacettepe University: Hacettepe Universitesi, TÜRKIYE

## Abstract

**Introduction:**

A strong body of evidence exists relating to inequality in general healthcare experience and outcomes for children and young people with learning disabilities and/or who are autistic compared to those without. Healthcare practitioners describe feeling less capable and confident to deliver care to children with learning disabilities, as well as having less capacity. However, there is little research specifically in cancer care that explores access and acceptability of provision for children with learning disabilities and/or those who are autistic. This is despite some cancers being more prevalent in syndromes associated with learning disabilities, for example Down’s Syndrome. We aim to explore the needs and experiences of children with/without learning disabilities and/or who are autistic and their families receiving cancer care. This inclusive study will provide evidence of whether, and what, inequity exists, for whom and why, generating evidence of what issues affect all children and young people receiving cancer care and what are particular to those with learning disabilities and/or who are autistic.

**Methods and analysis:**

We will use a transformative mixed methods design, comprising an individual staff and organisational level survey, retrospective case note review, ethnographic observations of clinical care, family and staff interviews, and participatory workshops. The ethnographer will follow and observe individual children and their families. We will use a ‘toolbox’ of creative participatory methods, including providing a co-designed research data collection journal to support elicitation of the child’s perspective. Data will be analysed using thematic analysis. The study will run from January 2025 to January 2026. The project is registered on ClinicalTrials.gov (Identifier: NCT06481527). Health Research Authority approval has been granted (REC Reference no. 24/LO/0410 | IRAS Project ID: 335623).

**Discussion:**

The mixed methods approach using survey and qualitative design will support a broad scope and in-depth understanding of the barriers and facilitators to inform equitable cancer care delivery for children with and without learning disabilities and/or who are autistic. Potential limitations are acknowledged. For example, resource constraints mean that the focus of the ethnography work package is within two hospital sites, which may limit broader comparisons and thematic development. Dissemination of findings will include papers specific to each work package. Recommendations for clinical practice will be developed, including staff training, healthcare planning and innovative solutions for improving the cancer care experience. These outputs will directly inform quality improvement from a local to national and international context in cancer care around children and young people with learning disabilities and/or who are autistic.

## Introduction

There is a long history of adults with learning disabilities experiencing poor treatment, care and discrimination in hospital. In 2007, for example, Mencap, a United Kingdom [UK] charity, published “Death by indifference” drawing attention to the cases of six people with learning disabilities who died unnecessarily in hospital and calling on the government to take “serious action” [1]. More recently evidence has shown that aspects of hospital care are also inequitable for children with learning disabilities, comparative to children and young people without learning disabilities, with staff reporting feeling less capable and confident to deliver care to those with learning disabilities, as well as having less capacity [2]. Similarly, a recent survey in the USA highlighted that children who are autistic are four times more likely to have unmet healthcare needs, when compared to children without disabilities, which was also higher than for children with other disabilities [3].

In relation to cancer care, there is limited research that explores access and acceptability of provision for underserved populations, such as children with learning disabilities and/or who are autistic. Recent work in adults with disabilities emphasises a lack of attitudinal and institutional preparation regarding cancer from healthcare professionals and institutions [4,5]. However, much less is known about how this inequality manifests with regards to experience and outcomes in paediatric cancer care [6]. Moreover, there is no known research that specifically seeks to understand the needs of children with learning disabilities and/or who are autistic, who also have cancer, and their families, so that services can be delivered equitably. This is despite the fact that certain cancer typologies are more prevalent for children and young people with learning disabilities compared to those without learning disabilities (Age 0–9: 0.7% vs. 0.1%; Age 10–17: 0.4% vs. 0.1%) [7]. For example, children and young people with Down’s Syndrome have greater risk of leukaemia [8] and poorer treatment outcomes [9,10] compared to children and young people without learning disabilities, including significant differences in treatment response and toxicity profiles. For other rare syndromes, such as Trisomy 13 and 18, population level data is limited but there is a similar pattern of an uneven distribution of certain cancer typologies [11,12]. Similarly, genetic pre-dispositions of different cancer profiles have been identified that are associated with diagnoses of Autism Spectrum Disorder (ASD) and other neurodevelopmental conditions [13–16], although globally incidence is equal between those who are autistic and those who are not. However, cancer mortality risk for those who are autistic is estimated to be nearly double compared to those who are not autistic, in a young adult population [17]. This difference arguably is related “to disparities in optimal cancer care” for reasons of limited screening access, delayed diagnosis and ineffective therapy for those patients who are also autistic [6]. Similar findings have been reported in adults with learning disabilities [18].

Children and young people with cancer who do not have learning disabilities and who are not autistic, report varying experiences of care and treatment. Their journey’s to cancer diagnosis are often protracted based on numerous factors, including clinicians overlooking signs and symptoms associated with possible diagnosis because of its rarity in this cohort [19], with reports of parents feeling they are not being listened too and sometimes having to engage in disputes and demand investigations [20]. When receiving cancer care, children and young people emphasise the importance of familiar environments, emotional support, and being informed about their treatment [21]. Older children in particular value involvement within interactions with clinicians and autonomy around decision-making, while younger children rely on their parents to greater extent [20–23]. How applicable such findings are to children and young people with learning disabilities or who are autistic is not known.

There are many complications of systemic anti-cancer treatment, which reduce quality of life: including nausea and vomiting; mucositis; constipation and diarrhoea; anaemia; anorexia; hair loss and febrile neutropenia [24,25]. Specific chemotherapy agents can cause particular morbidities, for example neuropathic pain from the use of vincristine [26]. Targeted treatments including surgery and radiotherapy can also have complications, such as radiation dermatitis [25]. We do know that one of the particular challenges faced in treating children with Down’s Syndrome and Leukaemia is balancing curative therapy against potential toxicities, requiring increased used of supportive care [8]. For example, chemotherapy tends not to be tolerated very well with a greater risk of side effects, particularly serious infections, which can make treatment more challenging [8]. Survival is also impacted. While Acute Myeloid Leukaemia in children with Down’s Syndrome is extremely sensitive to the effects of chemotherapy and responds very well to treatment, with trials suggesting a 5-year overall survival of more than 90%, this is not the same for Acute Lymphoblastic Leukaemia, where survival is lower than in the wider population [9,10]. Study based evidence for children who are autistic is less well developed. Nevertheless, we can anticipate challenges associated with some of the physical manifestations which overlap with common cancer or cancer treatment-related symptoms such as pain, sleep disturbances and fatigue, gastrointestinal (GI) problems or immune dysregulation [7]. These symptoms can be exacerbated by cancer-directed therapy or be misinterpreted. Furthermore, difficulties in communication in patients who are autistic can be an additional challenge to fully understanding their experience.

In addition to having intellectual impairment, children with learning disabilities may also have sensory and physical impairments adversely affecting speech, feeding and mobility [26]. They are almost twice as likely to report three or more health problems and more than four times as likely to suffer from a psychiatric disorder than children without learning disabilities [27]. Like some children who are autistic, their ability to understand information about hospital care and treatment may be severely limited, they may not be able to communicate their needs verbally, and may need additional support with all aspects of hospital life [28]. Those with challenging behaviour will find it particularly difficult, especially if care is not delivered in a therapeutic environment [29]. How these added complexities impact on the delivery of cancer care is unknown. The National Children’s Bureau [30] refer to children with learning disabilities as “an invisible group in society”, arguing that, “the more complex their needs, the more invisible they appear to become”, and as such those who also have cancer are at risk being doubly excluded.

This project seeks to build on previous work in which parents and children have shared experiences of discriminatory practices and of diagnostic overshadowing while asserting that they have not been properly listened to [28–30]. Within the field of paediatric cancer care, this project aims to address these research gaps and support implementation of ‘The learning disability improvement standards for NHS trusts’ [31,32], which encompasses: Respecting and protecting rights; Inclusion and engagement; Workforce; and Specialist learning disability service provision.

This evidence is needed to understand the context for making reasonable adjustments according to specific intellectual, emotional, social and physical needs. As personalised medicine is gaining popularity, we need a better understanding of what treatment outcomes matter to patients and families, particularly those with learning disabilities and/or who are autistic, for whom organisations have a legal responsibility to deliver reasonably adjusted care [33,34].

## Aim

Our aim is to understand the determinants of equitable cancer care for children and young people from diagnosis through to survivorship or palliative care, generating evidence of what issues affect all children and young people receiving cancer care and what are particular to those with learning disabilities and/or who are autistic.

## Objectives

### Phase 1 objectives

a. To compare healthcare professionals’ perceptions of their **capacity, capability and confidence** to deliver safe, holistic, individualised cancer care to children and young people with and without learning disabilities and/or autism

*We hypothesise that health professionals will perceive that they have less capacity, capability and confidence to deliver safe, holistic, individualised cancer care to those with learning disabilities and/or who are autistic and their families, compared to those without.*

b. To compare patient demographics, **care trajectory, symptom profiles and clinical** outcomes related to cancer care for children and young people with and without learning disabilities and/or autism

*We hypothesise that* c*hildren with learning disabilities and/or who are autistic*

- *will have a longer length of stay compared to children without learning disabilities and/or who are autistic and their families*
- *will have increased number and severity of side effects compared to children without learning disabilities and/or who are autistic and their families*

### Phase 2 objectives

a. To compare the needs and experiences of holistic, individualised cancer care and what outcomes matter most
b. To understand what constitutes a **safe and therapeutic environment** for the delivery of holistic, individualised cancer care to children and young people with and without learning disabilities and/or who are autistic
c. To understand the process of **transitioning from child to adolescent, and adolescent to adult healthcare** for children and young people with and without learning disabilities and/or who are autistic

### Phase 3 objectives

a. To identify and prioritise potential **innovative solutions** for improving experiences of cancer care
b. To co-design an open access **repository of information resources**

We anticipate that findings will enable healthcare professionals make better clinical decisions, helping to ensure that resources and interventions promoting equity are targeted to those who need them, how and when they need them. Furthermore, that the findings will be a catalyst for multiple improvements via increasing awareness, targeted training and further development of tailored interventions relevant to cancer care (Box 1).

**Box 1: Proposed targets for improvement**

1. Unmet needs
2. Adverse health outcomes/long-term emotional damage to children from poor practice
3. Numbers and type of complaints/clinical incidents
4. Patient/parent satisfaction and experience of care
5. Staff capability/confidence
6. Parental trust/confidence in care
7. Timely discharge
8. Financial cost to NHS/families
9. Therapeutic environment for delivery of cancer care
10. Transition experience from child to adolescent, or adolescent to adult services

Terminology and definition are crucial, for the terms used in this protocol see S1 File Supporting Information.

## Materials and methods

### Study design

A transformative, mixed methods case study design will be used. This approach focuses on underrepresented or marginalised populations [32,^34^], such as children with learning disabilities and/or who are autistic and their families who will represent each case. It “investigates contemporary phenomena in depth and within its real-life context” ([35], p.18) in this instance children receiving cancer care and treatment.

**Fig 1:**
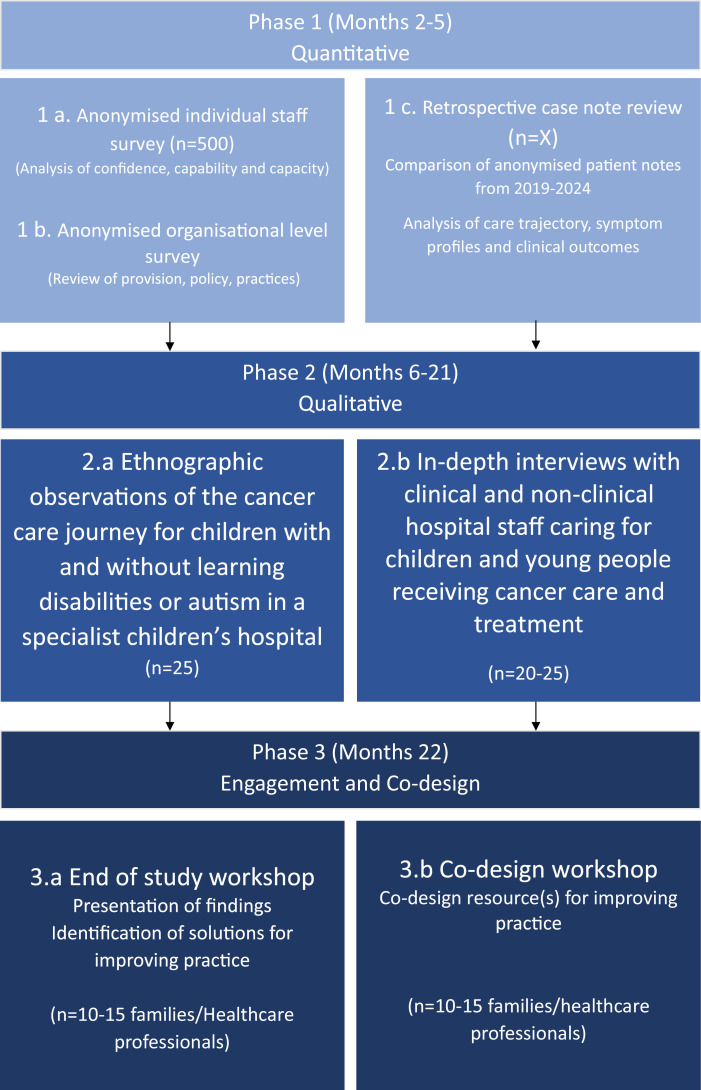
Study overview.

The study will involve three consecutive phases (Fig 1). Phase one is quantitative in nature and will comprise an individual and organisational hospital survey, and a retrospective case note review. Phase two is qualitative in nature and will comprise ethnographic observations of patients’ and families’ cancer journey as well as interviews with families and healthcare professionals. Ethnography is a recognised methodology for facilitating prolonged observations of the patient journey and enabling understanding of what people actually do, as well as what they say about what they hear and remember [36–40]. Use of this methodology is recognised as “creating a more efficient, more effective, more equitable and more humane health care system” ([41], p.2223). Phase three will involve two workshops with families and health care professionals to review the study findings, identify innovative solutions for improving care and equity, and co-design resources for use in practice.

### Public, patient involvement and stakeholder engagement

Public, patient involvement and stakeholder engagement are core to informing this study. We have established a parent advisory group (PAG) comprising eight parents whose children have previously received cancer care. These parents were drawn from existing hospital stakeholder groups, and further engagement with key stakeholder organisations, including the Down’s Syndrome Association and the Children & Young People Cancer Association (CCLG). The PAG will meet approximately three times per year, facilitated by the project coordinator and a clinical psychologist. In addition, a separate children and young persons’ advisory group has been made up, including children with and without learning disabilities and/or who are autistic. Both of these groups will support the study design development, including recruitment processes and participant facing materials.

### Setting

Children’s cancer care in the UK is delivered through a network of 21 tertiary centres, referred to as principal treatment centres [42] (PTC). The PTC retains overall responsibility for the cancer treatment plan, and for some PTCs defined aspects of care are delivered using a paediatric oncology shared care model, provided through Paediatric Oncology Shared Care Units [42] (POSCU). The phase 1 individual and organisational level survey will be conducted across the national network of PTCs and SCUs facilitated by the Children and Young people’s Cancer Association (CCLG). The retrospective case note review and phase two qualitative phase will be conducted primarily at one PTC, which partners in its care with another local site which will be involved as a secondary site. The service treats approximately 500 patients each year, accounting for approximately 28% of UK childhood cancer cases.

Phase 2 qualitative data collection will take place in a specialist children’s hospital on the cancer wards or outpatient settings, and other clinical areas relevant to delivery of cancer care.

## Methods and analysis

### Phase 1 quantitative

#### Hospital staff survey (1a).

A cross-sectional staff survey has been piloted and will be conducted across the UK in the first year of the project. It focuses on staff perceptions around their confidence to deliver equitable care for children with and without learning disabilities and/or who are autistic. The anonymised survey contains participant demographic questions, closed questions using mostly five-point Likert scales and free text questions. The survey will be distributed via the Children & Young People’s Cancer Association (CCLG) a national network and is open to all staff delivering cancer care in their role to children under 16 years. We will encourage those completing the survey to share with at least five colleagues across the multidisciplinary team, to create a snowballing sample. We estimate 500 responses will provide a robust data set with which to answer the research questions. Descriptive statistics will be used to characterise the sample. Composite variables will be computed to represent capability, capacity, confidence, safety and values separately for questions related to the two groups. Composite variables and individual questions, e.g., related to partnership working, environment, transition, parental burden and meeting needs, will be analysed using non-parametric statistics, comparing responses about children with learning disabilities or those who are autistic to those without. Comparison of responses from different professional groups and experience levels will be conducted using non-parametric statistics.

#### Organisational level survey (1b).

The organisational survey will be developed, piloted and distributed in year two of the project, following and informed by data collection and analysis from the individual healthcare professionals’ survey and early data collection and analysis from work package 2. The descriptive cross-sectional online survey will be anonymised and directed to clinical service level leads. The survey will map provision, policies and practices comprising a range of questions including focus around workforce, training, and service delivery models related to the delivery of cancer care to children with learning disabilities or who are autistic. Similar to work package 1b, the survey will be advertised through the CCLG network where organisational leads will be identified and made aware of the survey for completion. We estimate approximately 500 responses will provide a representative sample with sufficient scope to address the aims of this work package. The sample will be characterised using descriptive statistics. Composite variables and individual questions, e.g., related to provision, policies and practices will be analysed. Comparisons will be made between organisations using non-parametric statistics.

### Retrospective clinical case note review (1c)

The research team will work with the local digital data team to a) define the cohort specification, b) extract data according to pre-defined criteria such as patient demographics, co-morbidities, care episodes (e.g., length of stay, unplanned admissions), symptom profiles and treatment toxicity (e.g., pain, constipation, nausea and vomiting), and clinical outcomes (e.g., mortality, requirement for a bone marrow transplant, transfer to an end-of-life pathway).

The inclusion criteria will encompass children and young people who have received cancer care at the primary hospital research site between 1^st^ April 2019–31^st^ December 2023. This timeframe accounts for the implementation date of a new electronic patient record system within the hospital to the current time. A subsequent process will be undertaken to determine which of these patients have diagnoses of learning disabilities and/or ASD compared to those without. Within the digital environment, ICD-10 cancer related terms in oncology and haematology will be applied to establish the total case number. Against this case list, phrases relating to learning disabilities and ASD diagnoses will be applied to distinguish those cases with cancer and diagnoses of learning disabilities and/or ASD and those without. Data will be deidentified prior to analysis by the research team.

Data will cover standard structured clinical administrative data and free text clinical notes for all patients from the Electronic Patient Record (EPR) system. A data analyst will use Python and R to develop the pre-processing and exploratory data analysis code required in the initial pilot phase of the study. This analyst will develop a pipeline to wrangle the data into a format that is suitable for downstream analysis, including implementation of a text mining/search process and rule-based algorithm to identify and classify patients with and without learning disabilities and/or ASD diagnoses.

Statistical analysis will begin at a descriptive level to provide an overview of the different cohorts of children and young people with/without learning disabilities and/or ASD diagnoses. Further inferential statistics will be used to establish whether there are differences in outcomes associated with children having learning disabilities and/or ASD diagnoses using propensity score matching where appropriate, to reduce bias in the patient groups for comparison. The groups will be matched against a set of relevant factors. Resulting matched cohorts will be analysed to ascertain true differences between groups.

### Phase 2 qualitative

#### Ethnography (2a).

As is typical with Ethnographic studies, a purposeful sampling strategy will be used [40] to select participants according to pre-determined criteria. The sample size will be reflective of the expected time needed to build relationships with children with learning disabilities and engage them in the research process, as well as the knowledge that Ethnographic research yields large amounts of data. Early data analysis will iteratively inform further sampling to expand, refine and exhaust thematic development [43]. In this study, recruitment to data saturation will mean the corpus of data will be “large enough to capture a range of experiences but not so large as to be repetitious” ([44], p.193). Therefore, we will ensure that the full scope of the topic is explored in sufficient depth. We aim to recruit approximately 25 families with a ratio of 2:1 children with learning disabilities/ASD compared to those without.

The inclusion and exclusion criteria are outlined in Table 1.

**Table 1:** Eligibility criteria of participants for phase 2 data collection.

Participant	Inclusion criteria	Exclusion criteria
Children and young people	Receiving cancer care at the hospital research site, with/without learning disability, and/or who are autistic Ages 5–15	Within palliative care at the time of recruitment Subject to child safeguarding proceedings Outside of stated age range Where the family would prefer to maintain a complete non-disclosure approach to their child’s cancer diagnosis and feel their child’s participation may infringe on this request
Parents	Of children receiving cancer care in hospital research site If included for interview only, child’s age may be 0–15	Has mental health difficulties that would impair ability to participate effectively or create an unmanageable risk, e.g., psychosis

Data collection will include observations of the patient’s journey, clinical encounters, key ward events and key meetings between health professionals. Observation will be fully overt, with a visible encrypted digital recorder used to record conversations and written ethnographic fieldnotes will be kept [39]. Key recordings will be used to support more rapid write up of field notes and will be transcribed verbatim, providing an opportunity for conversation analysis [45].

Data collection will include informal discussions/creative sessions with children and parents. To ensure the research process is meaningful for children, irrespective of their communicative or intellectual ability, a toolkit of creative techniques suitable for children with learning disabilities and/or who are autistic will be available in line with the Mosaic Approach to research [46]. This could include resources such as Talking Mats, recordable speech bubbles, photography, objects of reference or play. Furthermore, a data collection research activity journal has been co-designed in conjunction with children, families and illustrators. The journal includes accessible and child appropriate activities for completion in between and during observations. The book is customisable with a mix of other fun activities such as writing, drawing and puzzles. Children will be able to complete as many or as few of the activities as they would like. The content of these entries will be revisited by the researcher with the children and young people and their parents to explore things that are meaningful to them, including about their identity, experience and reflections. We aim to understand aspects of children’s and young people’s multisensory experience within the ethnography [47,48]. Photography will be used by both children and young people and the researcher to capture important visual data relevant to the study aims [49]. Children and young people will use an instant camera to develop photographs that they will be able to stick into their activity book. Most important in the data collection process is being non-prescriptive and drawing on methods in accordance with children’s strengths and preferences, thereby enabling them to share their story in whatever way they are able and feel comfortable doing [50].

Families will be able to choose to participate in the ethnography to whatever level of involvement they prefer; for example, ranging from one-off 1−1 interviews to full involvement with observation and creative interviews. Our advisory groups have highlighted that siblings should not be overlooked and so they will be invited to participate where it is relevant to the study aims. In the circumstance that a child is isolated due to infection control precautions we will still offer the family the opportunity to participate. Where it is viable, discussions will take place between the researcher and the family via video conference calls and the child’s access to the creative resources/methods will be facilitated with the support of parents.

Phase 2 ethnography will include collection and analysis of material artifacts, such as a hospital passport or care plans, which will be explored for relevant information relating to the child’s strengths and needs in relation to their clinical care, with photos taken of these to illustrate findings. Documentary evidence will also involve access within the hospital electronic patient record for data extraction. Only data that are relevant to the study aims will be sought and accessed, for example the details of a reasonable adjustments flag, the clinical record of interaction between staff and family.

### Staff interviews (2b)

Semi-structured interviews will be undertaken with clinical and non-clinical hospital staff working with children receiving cancer care. The only eligibility criteria is that staff have experience of providing care to children undergoing cancer treatment at the hospital research site. Interviews will take place within a private setting of the participant’s choosing, face to face or online using video conference calls. A topic guide will use flexible and open-ended questions to explore the participants’ experience of receiving or providing cancer care in relation to the study aims and will also be informed by concurrent study findings. Interviews will last approximately 60 minutes, as guided by the participant. The interviews will be recorded on an encrypted digital recorder and transcribed verbatim by a third-party transcription company. Sensitive interviewing techniques will be used due to the risk of causing emotional trauma in view of the topic. A standard operating procedure will be used to manage emotional distress. An ongoing reflective diary will be kept to account for situational context, non-verbal observations during interviews and to promote self-awareness during all data collection activities [51].

Data from Phase 2 will be curated and managed using qualitative data analysis software NVIVO 14. Data will be analysed by the research team using Braun and Clark’s reflexive thematic analysis [43]. Hence, the analytical approach will be inductive, beginning with immersion (active reading) of the data using paper and pencil. Initial open coding will be undertaken before more focused coding towards developing higher level themes or organising concepts. Data collection will continue alongside analysis, meaning that it will be possible to explore themes of interest concurrently. For example, early interview data will provide a framework for potential ‘lines of enquiry’ to observe how what is said to happen occurs naturalistically in practice. Selected excerpts of clinical encounters/interactions will also be explored using conversation analysis to support the overarching analysis [52].

### Phase 3 staff and family workshops

Face to face staff and family workshops will be planned following data synthesis of the first two work packages. Previous participants, staff and families, will be brought together to hear about the study findings and explore innovative solutions for improving experiences and equity of cancer care (3a) and co-design [53,54] outputs for the open access repository of information (3b). Workshops will be designed in conjunction with the PPI Groups and will be co-facilitated in an accessible way with the research team. All participants will be provided with information about the workshops and will be reimbursed for their travel and time.

### Ethics

The study has received ethical approval from the London – Camden & Kings Cross Research Ethics Committee and the Health Research Authority (Rec no. 24/LO/0410).

The main ethical considerations relate to the involvement of vulnerable children and young people. Using the team’s extensive experience in this field, we will take a multifaceted approach to safeguard participants from harm according to beneficence principles. In accordance with the Nuffield Council on bioethics, an individualised approach will be taken to obtaining informed assent/consent with all children and young people according to age, learning and communication needs in partnership with their parents. For those with learning disabilities, adjustments will be made around assent/consent processes to support understanding and information retention. Information will be provided according to their preference; multiple options have been developed including resources that combine words, pictures and symbols, also a Stop Motion Animation using Playmobil, to explain what their participation will involve. We will be cognisant of how children and young people may express a wish to withdraw from the study and how to navigate the end of the research relationship. Consent processes involving adult participants (staff and parents) will follow a similar ethical code.

Ethnographic case studies require special consideration around maintaining participant confidentiality. Some families may feel that observation is too intrusive at some points. The child or young person and parents will be reminded throughout the study that they can ask the researcher to leave at any point and will ensure they know how they can easily indicate this (e.g., using signs). The researcher will remain vigilant to signs of participants’ distress/discomfort during research encounters and will proactively manage these situations with minimal disruption to all those involved. Partnership and rapport with families will be critical and the researcher’s awareness of each individual’s right to privacy and dignity during observation. The researcher will follow up with families and health professionals after any encounters in which the researcher was asked to leave, to determine whether support can be offered.

Preventing staff knowledge of family participation is impossible where data collection occurs on a ward, strict anonymisation procedures will be followed to ensure data confidentiality. Photographs and audio make an invaluable contribution to ethnography, but it is recognised that this element of the study must follow strict consenting and data protection procedures. Any photos or audio will be deleted or destroyed that inadvertently breach confidentiality or anonymity of others without their permission. All electronic data from patient records will be deidentified before extraction. Upon publication of findings, we will not report information that might compromise site or individual identity, for example relating to rare diagnoses or treatment, or location.

Throughout the project, all investigators will manage collection, storage, processing and disclosure of personal information in alignment with GDPR (2016) and Data Protection Act (2018) legislation. Unique identification numbers will be given to all participants, made known only to the research team. Demographic data will be kept to a minimum and will be stored separately to confidential and anonymised research data within a restricted access network drive, under a restricted access folder for the core research team.

## Discussion

This study aims to generate evidence of what matters to families within cancer care delivery. New insights will be developed relating to what constitutes a safe, holistic and therapeutic environment for delivering cancer care, and evidence of inequities in cancer care between children with and without learning disabilities and/or who are autistic, which includes an understanding of their experiences, symptom profiles and clinical outcomes.

The mixed methods approach using survey and ethnography design will support a broad scope and in-depth understanding of the barriers and facilitators to inform equitable cancer care for children with and without learning disabilities and/or who are autistic. Comparing these two groups of children and young people will strengthen our ability to identify inequity and understand where it exists, for whom and why. The use of creative, participatory and inclusive methods will facilitate the involvement of a diverse group of children and young people and their families and enable them to take part in an effective way to share what is meaningful to them in this study area. Study outputs will be co-constructed by the core research team, the steering committee, PAG and children’s and young people’s advisory group, following data synthesis and sharing of findings with other stakeholders in the third phase workshop. The proposed study outputs are outlined in the dissemination plan (Table 2). Findings will be used to inform learning disabilities’ accreditation standards, and have the potential to influence the focus of future Care Quality Commission (CQC) inspections, national learning disabilities benchmarking and the Oliver McGowan learning disability and autism mandatory staff training [55].

**Table 2:** Proposed study outputs.

Output	Content
**Recommendations development**	Recommendations for clinical practice, including staff training, healthcare planning, symptom management, and technological and innovative solutions for improving the cancer care experience.
**Accessible reports**	Participants will receive non-scientific reports in a range of accessible formats. An EasyRead version will be shared on relevant charity websites such as Down’s Syndrome Association, and National Autistic Society
**Open access publications and presentations**	Peer-reviewed manuscripts for open access journals will be produced alongside conference presentations at national and international conferences, such as International Society of Paediatric Oncology (SIOP)
**Online repository**	A repository of information/resources for healthcare professionals will be developed throughout the project and curated within a dedicated study website

Certain limitations could affect this study. It is possible that more hospital staff with a prior interest in the topic will complete the survey. This self-selection bias may limit the survey’s overall representativeness, but approach and recruitment strategies have been considered to address this challenge. For the ethnography, while it will offer an in-depth understanding of the research topic its intensive nature requires significant time and resources to execute. Resource constraints have meant that only two hospital sites will be included during the ethnography work package. However, this limitation will be mitigated with the mixed methods approach that will provide a broader understanding to inform the research aims.

In summary, this study will provide new understanding relating to the determinants of equitable cancer care for children and young people. By considering what issues affect all children and young people receiving cancer care and what are particular to those with learning disabilities and/or who are autistic we will fill a specific gap in the evidence.

## Supporting information

S1 File Terminology and definitions. (DOCX)
