# Supplementary material for: The seen and be heard study: A national mixed methods study to identify the barriers and facilitators to ensuring equitable cancer care for children with and without learning disabilities and/or who are autistic – Protocol Paper
Source: PLoS One. 2025 Nov 19;20(11):e0333020. doi: 10.1371/journal.pone.0333020 (PMC12629463; doi:10.1371/journal.pone.0333020)
Supplement: S1 File — (DOCX) [file pone.0333020.s001.docx]

## **Supplementary File 1**

## Terminology and Definitions

## Terminology

Within this protocol we refer to children with learning disabilities. There are many definitions of learning disability and outside the UK the preferred term is intellectual impairment. However, ‘Learning Disability’ is commonplace in policy documents and healthcare settings within the UK. The Department of Health defines learning disability as “a significantly reduced ability to understand new or complex information, to learn new skills (impaired intelligence), with a reduced ability to cope independently (impaired social functioning), which started before adulthood”, with a lasting effect on development (1, p.14). Level of impairment can be classified as mild, moderate, severe or profound (2).

Operationalising a theoretical definition of learning disabilities in practice can be problematic. Among very young children, only severe learning disability may be apparent and for some a formal diagnosis is never provided but they remain categorised as having ‘developmental delay’ or a ‘syndrome without a name’. For our purposes, a child will be included if any *one* of the following is documented in the medical notes:

a) A diagnosis of learning disability

b) A condition which is always accompanied by intellectual impairment, e.g. Down’s syndrome

c) Global developmental delay (GDD) in children aged over 5 years*

d) Has statement for Special Educational Needs or Education and Health Care Plan and parent confirms the child has learning disabilities

*Global Developmental Delay is defined as “significant delay (at least 2 standard deviations below the mean with standardized developmental tests) in at least two developmental domains in children under five years of age” (39, p.401) It includes children who are late in reaching their developmental milestones, which can be an indication of intellectual impairment, but may also be caused by factors such as inadequate stimulation, malnutrition, psychological and familial situations. In practice, some children will remain labelled as having GDD even when other causative factors have been ruled out and a diagnosis of learning disabilities is assumed by process of elimination - we want to make sure these patients are not excluded from taking part solely based on terminology

We apply the term ‘autistic people’ within this protocol as most people with ASD prefer to be identified in this way. The broader ASD diagnosis includes different levels of autism and conditions that previously were considered separate, e.g. Aspergers and Pervasive Developmental Disorder. Therefore, ASD is defined as lifelong neurodevelopmental and includes a diverse group of conditions. It is characterised by some degree of persistent difficulty with social interaction and communication and stereotypic (rigid and repetitive) behaviours, resistance to change or restricted interests (3-6). Other cognitive and behavioural characteristics include atypical patterns of activities, such as difficulty with transition from one activity to another, a focus on details and unusual reactions to sensations with possible emotional regulation difficulties (4,5). There has been an increase in prevalence of ASD diagnoses, partially attributed to increased awareness of the disorder at a younger age, as well as the 2013 revision of diagnostic criteria in the “Diagnostic and Statistical Manual of Mental Disorders”, which has led to an increase in diagnoses of milder presentations that need less adult support (5,7). To what extent, the typical pathway to an ASD diagnosis is disrupted by cancer care treatment is also an area of interest within this study.

1. HM Government. Valuing People - A New Strategy for Learning Disability for the 21st Century. In: Department of Health, editor. 2001.

2. World Health Organization. International Classification of Diseases, 11th Revision (ICD-11) 2022. Available from: <http://who.int>.

40.

3. Gillham JE, Carter AS, Volkmar FR, Sparrow SS. Toward a Developmental Operational Definition of Autism. Journal of Autism and Developmental Disorders. 2000;30(4):269-78.

4. NICE. Recognition, referral, diagnosis and management of adults on the autism spectrum England: National Institute for Health and Care Excellence; 2016.

5. NICE. Autism in adults: What is it? Clinical Knowledge Summaries 2020.

6. American_Psychiatric_Association. Diagnostic and statistical manual of mental disorders: DSM-5™, 5th ed. Arlington, VA, US: American Psychiatric Publishing, Inc.; 2013. xliv, 947-xliv, p.

7. Whitehouse AJO, Cooper MN, Bebbington K, Alvares G, Lin A, Wray J, et al. Evidence of a reduction over time in the behavioral severity of autistic disorder diagnoses. Autism Research. 2017;10(1):179-87.
